# Immune checkpoint inhibitors and their impact on liver enzymes and attenuation

**DOI:** 10.1186/s12885-022-10090-9

**Published:** 2022-09-20

**Authors:** Benjamin C. Park, Aaron X. T. Lee, Fei Ye, Isik Turker, Douglas B. Johnson

**Affiliations:** 1 Vanderbilt University School of Medicine, Nashville, TN USA; 2 Department of Medicine, Vanderbilt University Medical Center, 2220 Pierce Avenue, 777 Preston Research Building, Nashville, TN 3723 USA; 3 Department of Biostatistics, Vanderbilt University Medical Center, Nashville, TN USA

**Keywords:** Immune checkpoint inhibitors, Liver attenuation, Steatosis, Steatohepatitis, Immune related adverse events

## Abstract

**Background:**

Immune related adverse events impacting the liver are common from immune checkpoint inhibitor (ICI) therapy; however, there is little data regarding the subclinical impact of ICIs on liver inflammation. The study aims to determine whether ICI therapy affects liver attenuation and liver enzymes in melanoma patients with and without hepatic steatosis.

**Methods:**

A retrospective, cohort study was conducted of patients with advanced melanoma treated with ICI therapy who received serial PET-CT scans at the Vanderbilt University Medical Center (VUMC). Primary outcomes included: liver attenuation measured by PET-CT/non-contrast CT and liver enzymes. Hepatic steatosis was diagnosed by radiologists on clinical imaging.

**Results:**

Among 839 patients with advanced melanoma treated with ICIs, 81 had serial PET-CT scans approximately 12 months apart and long-term survival; of these 11 patients had pre-existing steatosis/steatohepatitis. Overall, ICI was not associated with significant increases in liver enzymes in all patients; modest decreases in liver enzymes were observed in patients with pre-existing steatosis/steatohepatitis. Similarly, liver attenuation did not change from baseline to post-treatment (58.44 vs 60.60 HU, + 2.17, *p* = 0.055).

**Conclusions:**

ICIs may not chronically affect liver enzymes or liver attenuation, a non-invasive measure of liver fat content and inflammation, in the general population or in those with pre-existing steatosis/steatohepatitis.

**Supplementary Information:**

The online version contains supplementary material available at 10.1186/s12885-022-10090-9.

## Introduction

Immune checkpoint inhibitors (ICIs) have known immune related adverse effects (irAEs) that present as autoimmune-like conditions in any organ system. The liver is a commonly affected organ (0.7–16%), primarily with “immune-mediated hepatitis (IMH).” [1, 2] The pathophysiology of hepatoxicity is likely from T-cell based autoimmunity to hepatocytes, which usually resolves with corticosteroid administration [2]. These events are diagnosed and graded based on liver enzymes, and severe events require ICI discontinuation [3, 4].

Beyond overt clinical events, ICIs may produce or worsen subclinical inflammation with more long-term effects [5]. Specifically, pre-existing inflammatory disorders may be exacerbated by ICIs, including atherosclerosis, steatohepatitis, and autoimmune disorders [6–10]. Since the worldwide incidence of NAFLD is approximately 25%, studying the impact of ICI therapy on this condition is a key need [11]. Steatosis (fat deposition in the liver) and steatohepatitis (steatosis with inflammation) represent a complex continuum of increasing inflammation and liver damage characterized by increased uptake or decreased clearance of fatty acids, triggering pro-inflammatory cytokines and infiltration of various immune cell types. Although largely reversible, progression of steatosis may result in steatohepatitis and further progression to fibrosis, cirrhosis, and hepatocellular carcinoma [12]. Within the context of ICIs, the blockade of the PD-1/L1 pathways has been associated not only with anti-tumor T cell responses, but with proatherogenic T-cell responses, the upregulation of genes involved in cholesterol synthesis, and a resulting increase in cellular cholesterol levels [6, 13]. Liver attenuation on CT scans is a useful adjunct in diagnosing diffuse hepatic diseases, particularly fat deposition [14]. Understanding these liver-based side effects of ICIs are necessary to better understand the risk–benefit profiles of treatment in these patients. However, challenges to studying this population include limited diagnostic assessments of disease status and the ability to follow long-term outcomes of NAFLD in patients with advanced malignancy.

Characterizing whether subclinical liver inflammation or fat deposition occurs in patients treated with ICI, or is exacerbated in patients with pre-existing steatosis/steatohepatitis has not been done. The aim of this study was to evaluate whether liver attenuation and liver enzymes were altered by ICI therapy in patients with long-term survival, and whether pre-existing steatosis/steatohepatitis worsened with ICI.

## Methods

Following institutional review board approval, a retrospective cohort study was conducted using the Vanderbilt University Medical Center (VUMC) melanoma database. As most CT scans are conducted with contrast, we primarily included patients with baseline and post-treatment PET-CT (non-contrast CT portion) and non-contrast CT scans. Contrasted CT scans were not used due to artifactual increases in liver attenuation. To remove confounding of cancer-progression related variables (e.g., cachexia), we included only patients with prolonged (≥ 2 year) survival and measured post-treatment BMI.

Data collection included: patient demographics, treatment characteristics, liver attenuation, and liver enzymes (Aspartate transaminase (AST), Alanine transaminase (ALT)). Baseline scans were defined as those obtained within 3 months of ICI initiation, and post-treatment as those obtained 10–12 months after ICI initiation. Absolute liver attenuation was measured in Hounsfield Units (HU) from a standardized region of interest using IDS7 PACS software (Sectra AB IDS7, 2020, version 22.1.21) on non-contrasted CT scans performed in isolation or as a component of PET-CT scans. Steatosis was defined as liver attenuation of < 40 HU as per standard definitions as measured by radiologists on the pre-treatment scan or scans during the past 24 months [15]. We identified two different cohorts of patients with pre-existing steatosis: 1) those with serial imaging as defined above (*n* = 11) and 2) those diagnosed as having steatosis by radiologists on pre-treatment scans but without serial non-contrasted CT imaging (*n* = 20). We assessed changes in liver attenuation in the first cohort, and changes in AST/ALT in both cohorts.

Continuous variables were reported with medians and frequencies (Number, %). Univariate and multivariate regression models were conducted for each primary outcome (liver attenuation, ALT, AST). Liver attenuation and liver enzyme measurements at different time points were compared with paired t-tests. Comparisons between subgroups were conducted with independent t-tests. Statistical significance for univariable tests was determined using FDR correction for multiple testing. Statistical analyses were conducted using R version 4.1.1.

## Results

Of 839 patients with advanced melanoma treated with ICI, 162 had at least 2-year overall survival (OS), including 81 with serial positron emission tomography (PET) or non-contrasted CT scans and 11 with pre-existing hepatic steatosis/steatohepatitis and liver metastases. Of these 81 patients with serial imaging and long-term survival, 54 were treated with single agent anti-PD-1/L1, 25 with ipilimumab and nivolumab, and 2 with ipilimumab monotherapy (Table S1). Of these, 9 patients developed IMH with treatment and 36 received steroids for any reason.

### Liver attenuation

**Fig. 1:**
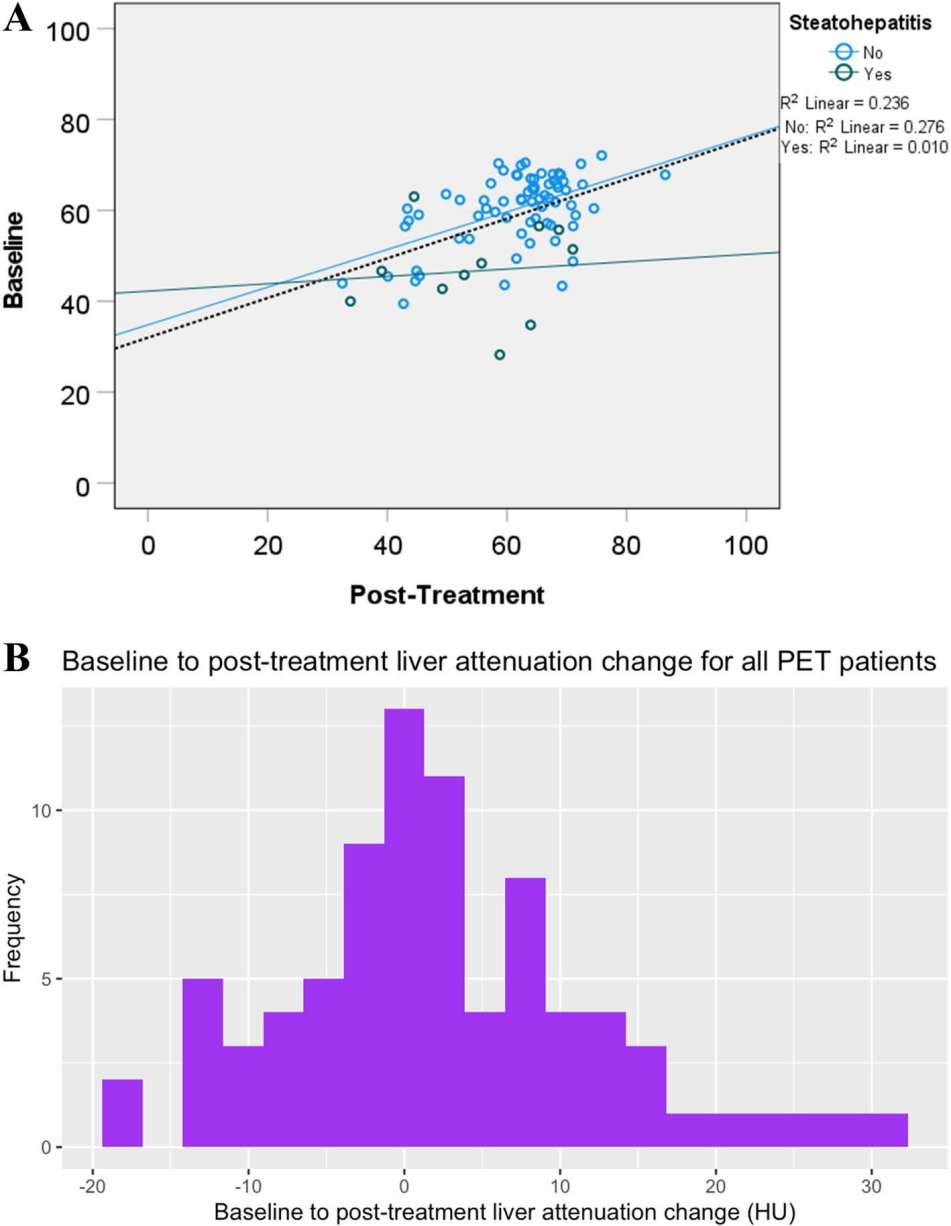
**A** Liver attenuation (HU) at baseline and post-ICI for patients with (green) and without pre-existing steatosis/steatohepatitis (blue). Black dotted line represents the overall line of best fit with an R-squared value of 0.236. Blue solid line represents the line of best fit for patients without steatosis/steatohepatitis with an R-squared value of 0.276. Green solid line represents the line of best fit for patients with steatosis/steatohepatitis with an R-squared value of 0.010. **B** Baseline to post-treatment liver attenuation change following ICI. Changes in liver attenuation generally follow a normal distribution, with few patients experiencing large changes. Most patients have little to no changes in liver attenuation with ICI

In the full cohort, there was no significant change in liver attenuation following ICI treatment (baseline vs post-treatment 58.4 vs. 60.6 HU, + 2.2, *p* = 0.055) with a non-significant trend toward increased attenuation/decreased fat deposition over time (Table 1, Fig. 1).

**Table 1:** Liver attenuation and liver enzymes by steatosis/steatohepatitis status

	**Overall (*n* = 81)**	**Steatosis/Steatohepatitis**		
**Liver attenuation (HU)**		**Yes (*n* = 11)**	**No (*n* = 70)**	***p value –* Between groups**
Baseline	58.44	46.67	60.29	< 0.001^*^
Post-treatment	60.60	54.83	61.51	0.460
∆ Post-treatment/Baseline	2.17	8.16	1.22	0.165
*p value –* ∆ Post-treatment/Baseline	0.055	0.103	0.247	-
	**Overall (*n* = 101)**	**Steatosis/Steatohepatitis**		
**Liver enzymes (AST)**		**Yes (*n* = 31)**	**No (*n* = 70)**	***p value –* Between groups**
Baseline	27.57	37.74	23.07	0.003^*^
Post-treatment	22.92	26.35	21.40	0.014
Last follow up	26.12	31.19	23.40	0.057
∆ Post-treatment/Baseline	-4.75	-11.39	-1.67	0.044
*p value –* ∆ Post-treatment/Baseline	0.005^*^	0.018	0.114	-
	**Overall (*n* = 101)**	**Steatosis/Steatohepatitis**		
**Liver enzymes (ALT)**		**Yes (*n* = 31)**	**No (*n* = 70)**	***p value –* Between groups**
Baseline	28.76	45.52	21.34	0.007*
Post-treatment	24.20	32.03	20.73	0.009*
Last follow up	25.28	28.13	24.03	0.332
∆ Post-treatment/Baseline	-4.75	-13.48	-0.61	0.093
*p value –* ∆ Post-treatment/Baseline	0.076	0.073	0.710	-

***Note*.** Statistical comparisons were conducted with independent t-tests for intergroup comparisons and paired t-tests for intragroup comparisons. *HU* Hounsfield unit

^*^Statistically significant at *p* < 0.05/*k* (*k* = number of tests performed) after Bonferroni correction for multiple testing

We then sought to determine whether high-risk groups would have greater changes with ICI treatment. Patients with baseline steatosis/steatohepatitis did not have statistically different changes in liver attenuation compared to those without these conditions (mean + 8.2 vs. + 1.2, *p* = 0.165), with a non-significant trend toward increased attenuation/decreased fat deposition on therapy. Unsurprisingly, these patients had lower baseline liver attenuation (46.7 vs. 60.3, *p* < 0.001) (Table 1). IMH during therapy (+ 4.5, *p* = 0.356; Table S2), any ICI-induced toxicity (+ 1.7, *p* = 0.296; Table S3), obesity (BMI ≥ 30) (+ 2.0, *p* = 0.378; Table S4), single agent therapy (+ 0.7, *p* = 0.579; Table S5), and steroid use (+ 1.7, *p* = 0.345; Table S6) were not associated with significant changes in liver attenuation from baseline to post-treatment. With the exception of single-agent vs. combination therapy, no other variable had significantly different changes in liver attenuation compared to their counterparts: IMH vs no IMH (4.5 vs. 1.9, *p* = 0.464), ICI-induced toxicity vs. no toxicity (1.7 vs. 2.9, *p* = 0.576), obese vs non-obese (2.0 vs. 2.3, *p* = 0.909), steroid use vs no steroid use (1.7 vs. 2.6, *p* = 0.694). Combination therapy, surprisingly, was associated with significant increases in liver attenuation following treatment compared with monotherapy (i.e. less fat deposition; + 5.5, *p* = 0.023) (Table S5). Multivariable analysis suggested that monotherapy (compared with combination) and higher baseline attenuation were associated with decreased liver attenuation whereas age, gender, obesity, steroid use, and baseline steatosis were not (Table 2).

**Table 2:** Multivariable regression model of predictors for changes in liver attenuation and liver enzymes

	**Overall (*n* = 81)**	
**Liver attenuation (HU)**	**∆**	***p***
Single agent therapy	-5.54 (-10.64, -0.44)	0.034*
Obesity (BMI ≥ 30)	1.57 (-5.93, 2.79)	0.475
Baseline steatosis/steatohepatitis	1.38 (-5.93, 2.79)	0.692
Steroid use	-1.50 (-6.06, 3.06)	0.514
Baseline liver attenuation	-0.42 (-0.68, -0.16)	0.002*
Age	0.06 (-0.10, 0.22)	0.459
Gender	1.56 (-2.95, 6.07)	0.493
	**Overall (*n* = 81)**	
**Liver enzymes (AST)**	**∆**	***p***
Single agent therapy	1.49 (-1.74, 4.72)	0.360
Obesity (BMI ≥ 30)	-0.23 (-3.01, 2.55)	0.871
Baseline steatosis/steatohepatitis	1.64 (-2.54. 5.82)	0.436
Steroid use	-3.21 (-6.08, -0.33)	0.030*
Baseline AST	-0.79 (-0.90, -0.68)	0.000*
Age	-0.03 (-0.13, 0.08)	0.633
Gender	0.64 (-2.23, 3.51)	0.659
	**Overall (*n* = 81)**	
**Liver enzymes (ALT)**	**∆**	***p***
Single agent therapy	6.46 (-0.77, 13.69)	0.079
Obesity (BMI ≥ 30)	2.86 (-3.29, 9.00)	0.358
Baseline steatosis/steatohepatitis	6.85 (-2.50, 16.20)	0.148
Steroid use	-1.36 (-7.72, 5.01)	0.673
Baseline ALT	-0.79 (-0.90, -0.67)	0.000*
Age	-0.20 (-0.44, 0.03)	0.091
Gender	4.51 (-1.88, 10.91)	0.164

***Note*.** Data are presented as mean change with the associated 95% confidence interval

*HU* Hounsfield unit

### Liver enzymes

To assess changes in liver enzymes, we combined the above cohort plus an additional 20 patients who were identified with baseline steatosis as diagnosed by radiologists on prior clinical imaging (*n* = 31 with steatosis, *n* = 101 total). These patients were not included in the initial analysis because they did not have serial PET-CT/non-contrast CT scans. Among the full cohort of patients, there was a significant decrease in AST from baseline to post-treatment (-4.8, *p* = 0.005) (Table 1). Baseline steatosis/steatohepatitis was associated with significant decreases in AST following treatment (-11.4, *p* = 0.018), although (unsurprisingly) higher liver enzymes at most timepoints (e.g. pre-treatment AST 37.7 vs. 23.1, *p* = 0.003; post-treatment AST 26.4 vs. 21.4, *p* = 0.014) (Table 1). ICI-induced toxicity was associated with significant decreases in AST (-5.1, *p* = 0.017) (Table S3). Obesity (BMI ≥ 30 vs. BMI < 30) was associated with significantly higher post-treatment ALT (28.0 vs. 19.6, *p* = 0.039), but no changes with therapy (-5.0, *p* = 0.09) (Table S4). Combination therapy was associated with significant decreases in AST (-4.8, *p* = 0.048) and ALT (-6.5, *p* = 0.007) (Table S5). Steroid use was associated with significantly lower post-treatment AST (19.8 vs. 23.9, *p* = 0.006) compared to no steroid use (Table S6). IMH and single-agent therapy were not associated with significant changes in liver enzymes. However, after controlling for covariates, only steroid use was associated with significant changes (decrease) in liver enzymes (Table 2).

Of the 31 patients with pre-existing steatosis, treatment included ipilimumab (*n* = 1), anti-PD-1 (*n* = 23), and ipilimumab and nivolumab (*n* = 7). Of these, only one patient developed IMH (3.2%), compared with 8 of 70 (11.4%) of patients lacking steatosis. This case occurred on pembrolizumab (grade 4) and resolved with high-dose steroids; no patients required mycophenolate mofetil. Ten patients had baseline liver enzymes above the upper limit of normal, potentially indicating steatohepatitis. Of note, 13 patients (42%) had transient increases in their liver enzymes during treatment of at least twice baseline, although none required steroids for resolution. Four of these patients received steroids for other reasons (pneumonitis, fevers, arthralgias, and adrenal insufficiency, respectively, the latter three only received low-dose prednisone dosed between 10-20 mg daily). Mean baseline, post-treatment, and last follow-up albumin levels were 4.3, 4.2, and 3.9 respectively. Last follow-up albumin levels were significantly decreased compared to post-treatment (-0.3, *p* = 0.004) and baseline albumin levels (-0.4, *p* < 0.001).

## Discussion

This study tested the hypothesis that ICIs might induce subclinical liver inflammation or exacerbate pre-existing steatosis/potential steatohepatitis [16–18]. Our analysis demonstrates that ICIs do not appear to worsen (decrease) liver attenuation or liver enzymes either in the general population or in patients with pre-existing risk factors. Provocatively, most trends that we observed were in the direction of improvement, particularly in patients with risk factors such as hepatic steatosis.

The relationship between pre-existing inflammatory conditions, including obesity-associated disorders with ICIs is complex. Beyond autoimmune diseases, ICIs may potentially affect the clinical course of chronic obesity associated inflammatory conditions including atherosclerosis [5, 19, 20]. Preclinical models have suggested that ICIs exacerbate obesity-associated inflammation [21, 22].

Somewhat surprisingly, patients with pre-existing steatosis/steatohepatitis had non-statistically significant increases in liver attenuation (suggesting, if anything, less infiltration/inflammation) and decreased (improved) liver enzymes following treatment. In contrast, long-term albumin levels were significantly decreased compared following treatment, although remained within normal limits. Moreover, other risk factors such as obesity were not associated with significant changes in liver attenuation and liver enzymes. IMH was also of particular interest as inflammation mediated immunogenic microenvironments (such as steatosis/steatohepatitis) may be also associated with increased T-cell activity and immune checkpoint expression, with uncertain influences on fat deposition and irAE risk [5, 23]. However, IMH incidence was rare in patients with baseline steatosis/steatohepatitis (1 of 31 patients) and was not associated with significant changes in liver attenuation or liver enzymes. Specifically, the single patient with high-grade IMH had normalization of liver attenuation and liver enzymes following steroids and prolonged follow up. Combination therapy increases both the incidence and severity of immune related adverse events, however we observed that this regimen was associated with increased liver attenuation [24].

Our findings suggest that ICI therapy appears to have a limited impact on liver function, although larger populations are needed to rule out small, subclinical impact. While this study is too small to suggest that ICI therapy improves steatosis/steatohepatitis, this seemingly incongruous finding should be studied further. However, this is the only study to date to begin unravelling the impact of ICIs within this population. Potential clinical explanations could include lifestyle changes associated with a cancer diagnosis (improved nutrition, exercise), progression from cancer (although only long-term survivors were included), or non-hepatic toxicities causing weight loss and nutrition changes (e.g. colitis). Alternatively, biological explanations might play some role as well; polarization of culpable macrophage populations would be one highly speculative explanations [25, 26]. ICIs have been reported to impact myeloid populations in the tumor microenvironment and in the context of irAEs [27]. However, the pathogenesis of this process, even outside the context of ICI therapy, is poorly understood, and further study is needed.

Of interest to clinical management, patients with pre-existing steatosis/steatohepatitis frequently experienced transient rises in their liver enzymes, usually early on therapy. Whether this represents a therapy-specific effect or simply routine fluctuations is not clear. However, only one patient experienced high-grade IMH; the remainder resolved or stabilized with observation and continued therapy. This suggests that clinicians should likely monitor patients with steatosis/ steatohepatitis closely with low-grade liver enzyme increases, and reserve steroids for higher-grade events.

### Limitations

Limitations include the single-center nature, small sample size, and inclusion of solely melanoma patients. Larger multi-institution studies are necessary to validate these findings. Further, more sensitive imaging modalities (e.g. MRI) and longer term analyses may also provide additional insights. Longer-term studies are also needed to rule out delayed progression from steatosis to steatohepatitis or cirrhosis. Finally, assessment of cytokine levels and other inflammatory markers over time would be useful in patients with NAFLD receiving ICIs.

## Conclusions

ICIs were not associated with subclinical changes in liver attenuation or liver enzymes following therapy. This supports ICI use in patients with pre-existing steatosis/steatohepatitis. However, larger studies are necessary to confirm these results and to further elucidate the role of liver attenuation in ICI-induced toxicity diagnosis and management.

## Supplementary Information

**Additional file 1.**

## Data Availability

We do not wish to share our data at this time. All data generated or analyzed during this study are included in this published article [and its supplementary information files].
