# Supplementary material for: Immune checkpoint inhibitors and their impact on liver enzymes and attenuation
Source: BMC Cancer. 2022 Sep 20;22:998. doi: 10.1186/s12885-022-10090-9 (PMC9487144; doi:10.1186/s12885-022-10090-9)
Supplement: Supplementary file 1 — Additional file 1. [file 12885_2022_10090_MOESM1_ESM.docx]

**ONLINE-ONLY SUPPLEMENTS**

**TABLE LEGEND**

**Table S1 – Patient Demographics and Treatment Characteristics (PET/CT)**

**Table S2 – Liver Attenuation and Liver Enzymes for IMH Subgroup**

**Table S3 – Liver Attenuation and Liver Enzymes for Any ICI-Induced Toxicity Subgroup**

**Table S4 – Liver Attenuation and Liver Enzymes for Obesity Subgroup**

**Table S5 – Liver Attenuation and Liver Enzymes for Single vs Combination Therapy Subgroups**

**Table S6 – Liver Attenuation and Liver Enzymes for Steroid Treatment Subgroup**

**Table S1. Patient Demographics and Treatment Characteristics (PET/CT)**

| **Characteristic** | **Number of patients (%)** |
| --- | --- |
| **Age (median)** | 60.00 |
| **Sex (male)** | 55 (67.9%) |
| **Initial BMI (median)** | 29.10 |
| **Post-treatment BMI (2 years, median)** | 29.10 |
| **Overall survival (median; years)** | 3.42 |
| **Initial stage at start of treatment** | |
| M1a | 26 (32.1%) |
| M1b | 14 (17.3%) |
| M1c/d | 20 (24.7%) |
| Other (IIB-IIID) | 21 (25.9%) |
| **ICI treatment** | |
| Pembrolizumab | 39 (48.1%) |
| Ipilimumab + Nivolumab | 25 (30.9%) |
| Nivolumab | 13 (16.0%) |
| Ipilimumab | 2 (2.5%) |
| Atezolizumab | 2 (2.5%) |
| **Any toxicity** | 48 (59.3%) |
| **Steroid treatment** | 36 (44.4%) |
| **Liver toxicity** | 9 (11.1%) |
| **Steatosis/Steatohepatitis** | 11 (13.6%) |
| **Liver Metastasis** | 11 (13.6%) |
| **BMI** |  |
| <30.0 | 49 (60.5%) |
| ≥30.0 | 32 (39.5%) |

**Table S2. Liver Attenuation and Liver Enzymes for IMH Subgroup**

|  | **IMH** | | |
| --- | --- | --- | --- |
| **Liver attenuation (HU)** | **Yes (n=9)** | **No (n=72)** | ***p value –* Between groups** |
| Baseline | 57.30 | 58.58 | 0.701 |
| Post-treatment | 61.79 | 60.46 | 0.718 |
| ∆ Post-treatment/Baseline | 4.49 | 1.88 | 0.464 |
| *p value –* ∆ Post-treatment/Baseline | 0.356 | 0.100 | - |
| **Liver enzymes (AST)** | **Yes (n=9)** | **No (n=72)** | ***p value –* Between groups** |
| Baseline | 28.56 | 24.86 | 0.442 |
| Post-treatment | 23.11 | 21.94 | 0.626 |
| Last follow up | 22.33 | 24.64 | 0.671 |
| ∆ Post-treatment/Baseline | -5.44 | -2.92 | 0.549 |
| *p value –* ∆ Post-treatment/Baseline | 0.113 | 0.045 | - |
| **Liver enzymes (ALT)** | **Yes (n=9)** | **No (n=72)** | ***p value –* Between groups** |
| Baseline | 26.44 | 26.43 | 0.999 |
| Post-treatment | 27.11 | 22.36 | 0.680 |
| Last follow up | 25.22 | 25.64 | 0.956 |
| ∆ Post-treatment/Baseline | 0.67 | -4.07 | 0.593 |
| *p value –* ∆ Post-treatment/Baseline | 0.948 | 0.161 | - |

**Note.** Statistical comparisons were conducted with independent t-tests for intergroup comparisons and paired t-tests for intragroup comparisons. HU = Hounsfield unit.

*Statistically significant at p<0.05/k (k=number of tests performed) after Bonferroni correction for multiple testing

**Table S3. Liver Attenuation and Liver Enzymes for ICI-Induced Toxicity Subgroup**

|  | **Any toxicity** | | |
| --- | --- | --- | --- |
| **Liver attenuation (HU)** | **Yes (n=48)** | **No (n=33)** | ***p value –* Between groups** |
| Baseline | 58.94 | 57.71 | 0.562 |
| Post-treatment | 60.59 | 60.63 | 0.985 |
| ∆ Post-treatment/Baseline | 1.65 | 2.92 | 0.576 |
| *p value –* ∆ Post-treatment/Baseline | 0.296 | 0.069 | - |
| **Liver enzymes (AST)** | **Yes (n=48)** | **No (n=33)** | ***p value – Between groups*** |
| Baseline | 26.69 | 23.21 | 0.258 |
| Post-treatment | 21.63 | 22.73 | 0.472 |
| Last follow up | 26.29 | 22.16 | 0.245 |
| ∆ Post-treatment/Baseline | -5.06 | -0.48 | 0.087 |
| *p value – ∆ Post-treatment/Baseline* | 0.017 | 0.672 | - |
| **Liver enzymes (ALT)** | **Yes (n=48)** | **No (n=33)** | ***p value – Between groups*** |
| Baseline | 29.63 | 21.79 | 0.251 |
| Post-treatment | 23.08 | 22.61 | 0.895 |
| Last follow up | 29.95 | 20.88 | 0.042 |
| ∆ Post-treatment/Baseline | -6.54 | 0.82 | 0.193 |
| *p value – ∆ Post-treatment/Baseline* | 0.152 | 0.622 | - |

**Note.** Statistical comparisons were conducted with independent t-tests for intergroup comparisons and paired t-tests for intragroup comparisons. HU = Hounsfield unit.

*Statistically significant at p<0.05/k (k=number of tests performed) after Bonferroni correction for multiple testing

**Table S4. Liver Attenuation and Liver Enzymes for Obesity Subgroup**

|  | **Obesity** | | |
| --- | --- | --- | --- |
| **Liver attenuation (HU)** | **<30 (n=49)** | **≥30 (n=32)** | ***p value –* Between groups** |
| Baseline | 59.76 | 56.41 | 0.146 |
| Post-treatment | 62.04 | 58.40 | 0.123 |
| ∆ Post-treatment/Baseline | 2.28 | 1.99 | 0.909 |
| *p value –* ∆ Post-treatment/Baseline | 0.054 | 0.378 | - |
| **Liver enzymes (AST)** | **<30 (n=49)** | **≥30 (n=32)** | ***p value –* Between groups** |
| Baseline | 23.35 | 28.22 | 0.113 |
| Post-treatment | 21.33 | 23.22 | 0.218 |
| Last follow up | 23.45 | 25.81 | 0.498 |
| ∆ Post-treatment/Baseline | -2.02 | -5.00 | 0.271 |
| *p value –* ∆ Post-treatment/Baseline | 0.059 | 0.097 | - |
| **Liver enzymes (ALT)** | **<30 (n=49)** | **≥30 (n=32)** | ***p value –* Between groups** |
| Baseline | 21.43 | 34.09 | 0.126 |
| Post-treatment | 19.55 | 28.00 | 0.039 |
| Last follow up | 23.82 | 28.31 | 0.349 |
| ∆ Post-treatment/Baseline | -1.88 | -6.09 | 0.542 |
| *p value –* ∆ Post-treatment/Baseline | 0.191 | 0.370 | - |

**Note.** Statistical comparisons were conducted with independent t-tests for intergroup comparisons and paired t-tests for intragroup comparisons. HU = Hounsfield unit.

*Statistically significant at p<0.05/k (k=number of tests performed), Bonferroni corrected for multiple testing

**Table S5. Liver Attenuation and Liver Enzymes for Single vs Combination Therapy Subgroups**

|  | **Single or Combination Therapy** | | |
| --- | --- | --- | --- |
| **Liver attenuation (HU)** | **Single (n=56)** | **Combination (n=25)** | ***p value –* Between groups** |
| Baseline | 58.68 | 57.90 | 0.733 |
| Post-treatment | 59.36 | 63.40 | 0.105 |
| ∆ Post-treatment/Baseline | 0.68 | 5.50 | 0.045 |
| *p value –* ∆ Post-treatment/Baseline | 0.579 | 0.023 | - |
| **Liver enzymes (AST)** | **Single** | **Combination** | ***p value –* Between groups** |
| Baseline | 25.34 | 25.12 | 0.947 |
| Post-treatment | 22.84 | 20.36 | 0.126 |
| Last follow up | 24.18 | 24.84 | 0.858 |
| ∆ Post-treatment/Baseline | -2.50 | -4.76 | 0.431 |
| *p value –* ∆ Post-treatment/Baseline | 0.126 | 0.048 | - |
| **Liver enzymes (ALT)** | **Single** | **Combination** | ***p value –* Between groups** |
| Baseline | 27.79 | 25.64 | 0.875 |
| Post-treatment | 24.57 | 19.12 | 0.155 |
| Last follow up | 24.57 | 27.88 | 0.515 |
| ∆ Post-treatment/Baseline | -2.21 | -6.52 | 0.475 |
| *p value –* ∆ Post-treatment/Baseline | 0.570 | 0.007* | - |

**Note.** Statistical comparisons were conducted with independent t-tests for intergroup comparisons and paired t-tests for intragroup comparisons. HU = Hounsfield unit.

*Statistically significant at p<0.05/k (k=number of tests performed), Bonferroni corrected for multiple testing

**Table S6. Liver Attenuation and Liver Enzymes for Steroid Use Subgroup**

|  | **Steroid** | | |
| --- | --- | --- | --- |
| **Liver attenuation (HU)** | **Yes (n=36)** | **No (n=45)** | ***p value –* Between groups** |
| Baseline | 59.73 | 57.40 | 0.249 |
| Post-treatment | 61.40 | 59.96 | 0.539 |
| ∆ Post-treatment/Baseline | 1.67 | 2.56 | 0.694 |
| *p value –* ∆ Post-treatment/Baseline | 0.345 | 0.085 | - |
| **Liver enzymes (AST)** | **Yes** | **No** | ***p value –* Between groups** |
| Baseline | 24.31 | 26.04 | 0.568 |
| Post-treatment | 19.80 | 23.89 | 0.006* |
| Last follow up | 25.08 | 23.82 | 0.714 |
| ∆ Post-treatment/Baseline | -4.50 | -2.16 | 0.379 |
| *p value –* ∆ Post-treatment/Baseline | 0.071 | 0.126 | - |
| **Liver enzymes (ALT)** | **Yes** | **No** | ***p value –* Between groups** |
| Baseline | 27.53 | 25.56 | 0.771 |
| Post-treatment | 20.81 | 24.56 | 0.295 |
| Last follow up | 26.94 | 24.51 | 0.607 |
| ∆ Post-treatment/Baseline | -6.72 | -1.00 | 0.307 |
| *p value –* ∆ Post-treatment/Baseline | 0.262 | 0.536 | - |

**Note.** Statistical comparisons were conducted with independent t-tests for intergroup comparisons and paired t-tests for intragroup comparisons. HU = Hounsfield unit.

*Statistically significant at p<0.05/k (k=number of tests performed), Bonferroni corrected for multiple testing
